# *Listeria innocua* Dps as a nanoplatform for bioluminescence based photodynamic therapy utilizing *Gaussia princeps* luciferase and zinc protoporphyrin IX

**DOI:** 10.1016/j.nano.2019.04.008

**Published:** 2019-08

**Authors:** Ali W. Al-Ani, Lei Zhang, Lenny Ferreira, Lyudmila Turyanska, Tracey D. Bradshaw, Neil R. Thomas

**Affiliations:** a Centre for Biomolecular Sciences, School of Chemistry, The University of Nottingham, Nottingham, UK; b School of Physics & Astronomy, The University of Nottingham, University Park, Nottingham, UK; c and School of Chemistry, University of Lincoln, UK; d Centre for Biomolecular Sciences, School of Pharmacy, The University of Nottingham, University Park, Nottingham, UK

**Keywords:** Bioluminescence resonance energy transfer, *Gaussia princeps* luciferase, *Listeria innocua* DNA binding protein, Photodynamic therapy, Zinc (II) protoporphyrin IX

## Abstract

*Listeria innocua* DNA binding protein from starved cells (LiDps) belongs to the ferritin family and provides a promising self-assembling spherical 12-mer protein scaffold for the generation of functional nanomaterials. We report the creation of a *Gaussia princeps* luciferase (Gluc)-LiDps fusion protein, with chemical conjugation of Zinc (II)-protoporphyrin IX (ZnPP) to lysine residues on the fusion protein (giving Gluc-LiDps-ZnPP). The Gluc-LiDps-ZnPP conjugate is shown to generate reactive oxygen species (ROS) *via* Bioluminescence Resonance Energy Transfer (BRET) between the Gluc (470-490 nm) and ZnPP. *In vitro*, Gluc-LiDps-ZnPP is efficiently taken up by tumorigenic cells (SKBR3 and MDA-MB-231 breast cancer cells). In the presence of coelenterazine, this construct inhibits the proliferation of SKBR3 due to elevated ROS levels. Following exposure to Gluc-LiDps-ZnPP, migration of surviving SKBR3 cells is significantly suppressed. These results demonstrate the potential of the Gluc-LiDps-ZnPP conjugate as a platform for future development of an anticancer photodynamic therapy agent.

## Graphical Abstract

A ‘self-illuminating’ nanoparticle for photodynamic therapy has been created based on the Dps protein nanocage. This converts bioluminescence from an excited *Gaussia* luciferin into reactive oxygen species (ROS) *via* a bioluminescence resonance energy transfer (BRET) process with zinc protoporphyrin. The biological activity of this agent with a variety of cancerous and healthy cell lines is presented.

**Figure:**
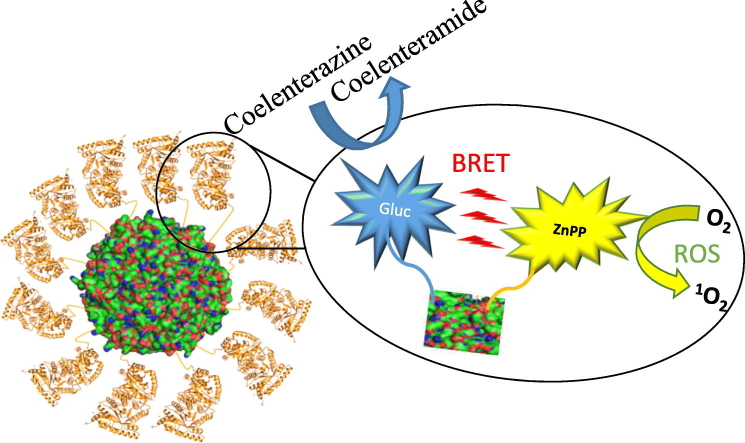

Photodynamic therapy (PDT) is an emerging therapeutic modality for non-invasive and tissue selective treatment of cancer and other diseases.^1^ Success of the treatment relies on excitation of a non-toxic photosensitizer in the presence of oxygen to generate singlet oxygen (*via* a type II photochemical reaction) or other intra-cellular reactive oxygen species (ROS) (*via* a type I photochemical reaction).^2^ ROS are strong oxidizing agents which attack DNA, proteins and lipids causing irreversible damage to cells.^3^ Several PDT agents have already been used for clinical treatment of age-related macular degeneration^4^ and proposed for the treatment of various carcinomas.^5^, ^6^, ^7^ Because the photosensitizers require light for activation, most of the clinical studies against cancer have focused on treating tumors that are on or just below the surface of the skin or in the exterior lining of internal organs and cavities, given the light penetration at the wavelengths required is normally less than 1 cm due to absorption and scattering caused by the tissue.^8^ Strategies for overcoming this have recently been reviewed.^9^, ^10^, ^11^ Selective targeting of PDT agents to diseased tissues is required to reduce the collateral damage to healthy cells,^12^ however achieving specificity and selectivity in PDT, beyond controlling the area illuminated, remains challenging.

Bioluminescence Resonance Energy Transfer (BRET) offers one option for avoiding the need for an external light source and allowing localized treatment of tumors deeper in a patient's body.^13^ The efficiency of a BRET pair relies on overlap of the bioluminescent emission generated by luciferase enzymes or photoproteins and the absorption of the photosensitizer which must be located within 10-100 nm of each other. Within the luciferase family, *Gaussia* luciferase (Gluc) from the marine copepod *G. princeps* offers several advantages over other luciferases including its low molecular weight (19.9 kDa),^14^ and bright peak bioluminescence at 480 nm.^15^ Also, unlike other luciferases there are, no requirement for ATP or any other cofactors,^16^ only the coelenterazine (luciferin) substrate to generate light. It has been demonstrated that luciferases and luciferins are non-toxic to many different cell types.^17^, ^18^

Among various photosensitizers, zinc (II) protoporphyrin IX (ZnPP) has attracted significant interest as it has shown the ability to generate singlet oxygen upon illumination with visible light with a quantum yield of 0.91.^19^, ^20^ A potential scaffold for the donor-acceptor BRET pair could be provided by *Listeria innocua* DNA binding protein from starved cells (LiDps). This protein from the ferritin family, a so-called ‘mini-ferritin’ is composed of twelve subunits (each 19 kDa) that assemble into a spherical cage with an external diameter 9 nm and hollow central cavity with a diameter of 4.5 nm,^21^, ^22^ which in addition, could be used for delivery of therapeutic agents. This scaffold has a number of advantages: it is readily expressed in *E. coli* and has no cysteine residues. The self-assembly of the scaffold is pH and [urea] sensitive with the capsule disassociating at pH 2.0 allowing differently functionalized subunits to be combined to give capsules with a variety of stoichiometries,^23^, ^24^ it allows proteins to be fused to both its N- and C-termini which are on the exterior of the protein capsule without affecting capsule assembly,^25^ fusion of small proteins such as *Gaussia* luciferase to the Dps capsule prevents them from being removed by glomerular filtration in the kidneys significantly extending their circulation time as the capsule is above the ~70 kDa glomerular filtration threshold cut-off,^26^ this protein scaffold unlike many polymeric or inorganic nanoparticle based systems is also fully biodegradable removing any concerns of persistence within the body. Dps is an iron (II) scavenging protein that possesses ferroxidase activity. It is thought that their natural role is to prevent DNA from Fenton mediated oxidative stress in *L. innocua* as it catalyzes iron(II) to iron(III) oxidation in the presence of hydrogen peroxide reducing the production of hydroxyl radicals that can damage DNA.^27^ The ferroxidase activity is lost in the absence of iron or in the H31G-H43G and H31G-H43G-D58A mutants.^28^

To date, a very small number of other BRET-based PDT agents have been reported: Rose Bengal sensitiser conjugated to *Renilla* luciferase,^13^ *Renilla* luciferase-immobilized quantum dots-655 (QD-RLuc8) in combination with *meta*-tetra(hydroxyphenyl)chlorin (m-THPC) (temoporfin/Foscan)-loaded micelles^29^, ^30^ and *Renilla* luciferase (Rluc8) conjugated carboxylated 655-nm quantum dots and chlorin e6.^31^ In an alternative approach, the luciferase gene can be transfected into the cells and the sensitizer added separately: A firefly luciferase-based system for the excitation of Rose Bengal in NIH 3T3 murine fibroblasts was described by Theodossiou in 2003,^32^ although these results have been contested.^33^, ^34^ This approach is limited by the need to deliver the gene for the luciferase selectively to the tumor cells only, which is currently not possible in the clinic, however application of chemiluminescence and bioluminescence in PDT continues to attract considerable attention.^35^

Here we report the development of a new biocompatible multifunctional nanoplatform with potential applications as a ‘self-illuminating’ PDT agent. Gluc has been genetically fused to the N-terminal of LiDps subunits and the lysine residues on the resulting fusion protein surface have been conjugated with ZnPP. The Gluc-LiDps-ZnPP conjugate operates *via* BRET where the coelenterazine is excited by Gluc and transfers its energy to the photosensitizer (ZnPP) resulting in the production of ROS. *In vitro*, Gluc-LiDps-ZnPP is efficiently sequestered by tumor cells and, in the presence of the coelenterazine, inhibits the proliferation of breast cancer cells SKBR3 due to ROS generation in a self-contained photodynamic therapy system. Remarkably, following exposure to Gluc-LiDps-ZnPP, the cell migration of surviving cells is also significantly suppressed. These results demonstrate the realistic potential of the Gluc-LiDps-ZnPP nanoconstruct as a starting point for the development of an anticancer PDT agent.

## Methods

### Cloning of Gluc-LiDps

Both LiDps and Gluc genes were constructed in pJexpress vectors with the LiDps gene being a gift from Dr. Phil Hill, University of Nottingham and Gluc being a synthetic gene (DNA2.0) having been codon optimized for *E. coli* protein expression. An *E. coli* vector was constructed by sub-cloning the Gluc gene from plasmid pJexpress414 into pJexpress411 which contained the LiDps gene. The Gluc gene was fused to the N-terminal of LiDps *via* a DNA sequence encoding a flexible pentaglycine linker and thrombin cleavage site peptide (TGGGGGGLVPRGS). To assist purification, DNA encoding a hexa-His-tag was placed at the N-terminal of the Gluc protein. The complete amino acid sequence of the Gluc-LiDps construct is given in the supplementary information.

### Expression and purification of Gluc-LiDps

Gluc-LiDps expression was induced by IPTG (1 mM) (Fisher Scientific) overnight (~16 h) in cultures of *E. coli* BL21 (DE3) carrying the pJexpress411:Gluc-LiDps plasmid and grown in Luria Bertani (LB) medium supplemented with kanamycin (50 μg /mL) (Apollo Scientific) at 30 °C. Cells were harvested by centrifugation at 2500 ×*g* for 25 minat 4 °C, resuspended in 40 mL buffer (20 mM Tris pH 8.3 (Fisher Scientific), 10 mM imidazole (Sigma Aldrich)) with 1 mM phenyl methyl sulfoxide (PMSF) (Sigma Aldrich) and lysed by sonication. After centrifugation at 22,000 ×*g* for 25 min at 4 °C, the resulting pellet was resuspended in the IMAC binding buffer (20 mM Tris pH 8.3, 10 mM imidazole, 4 M urea (Fisher Scientific)) and centrifuged again at 35,000 ×*g*. The protein solution was loaded on a 5 mL nickel column (GE Healthcare) and washed 3× with the column volume of binding buffer. Gluc-LiDps was then eluted using a linear gradient of increasing imidazole concentration up to 500 mM. Following purification, the Gluc-LiDps was refolded when dialyzed against two changes of phosphate buffer (2 L, 20 mM Na_2_HPO_4_ pH 8.3 (Fisher Scientific)) using 6-8 K MWCO dialysis tubing (Spectrum Laboratories). The Gluc-LiDps fractions were identified using SDS-gel electrophoresis. The fractions containing Gluc-LiDps were pooled and loaded on a size exclusion column (Superdex 75 10/300 GL). Eluted fractions were analyzed using SDS PAGE and the Gluc-LiDps concentration was determined by absorbance measurements (extinction coefficient = 24,575 M cm^−1^) using a NANO Drop A-1000 spectrophotometer. The yield of protein refolding was 5 mg per 1 L of culture.

#### Spectrophotometric measurements

Serial dilutions of Gluc-LiDps and Gluc-LiDps-ZnPP with final concentrations of 0.5, 1, 5, and 10 μg/mL were prepared using phosphate buffer (20 mM Na_2_HPO_4,_ pH 8.3). The samples were mixed with 2, 4, or 6 μg/mL of coelenterazine in the presence and absence of 0.1% (w/v) SDS in a white 96 well opaque plate (PerkinElmer) and were analyzed using a microplate reader (En Vision 2104 Multilabel Reader). Coelenterazine (Nanolight Technologies, Arizona USA) was dissolved in methanol (0.5 mg per 20 μL) and added to 118 μL of phosphate buffer. Coelenterazine stock solutions (10 mM) were stored at −80 °C.

### Chemical modification of Gluc-LiDps subunits with ZnPP

A general procedure^36^ was followed. Briefly, the ZnPP-NHS ester was prepared by mixing 50 μL of ZnPP (40 mM) in DMSO solution with 8 μL of 1-(3-dimethylaminopropyl)-3-ethylcarbodiimide hydrochloride (EDC) (500 mM) and then 8 μL of *N*-hydroxysuccinimide (NHS) (500 mM) being added (ZnPP:EDC:NHS = 1:2:2). The resulting solution was stirred for 15 min and centrifuged at 5000 ×*g* for 5 min to remove the soluble isourea by-product and excess reagents. The purified Gluc-LiDps fusion protein 800 μL (0.7575 mM) (contains 30 lysine residues + N-terminal amine per subunit) was incubated with 200 μL activated ZnPP-NHS ester (30.3 mM) at a molar ratio of 1:10 of Gluc-LiDps to ZnPP-NHS. After 24 h at room temperature, the unreacted materials were removed by dialysis (2 × 2 L) of phosphate buffer (20 mM Na_2_HPO_4,_ pH 8.3) using 6-8 K MWCO dialysis tubing (Spectrum Laboratories). Samples were then filtered through (C18) Zip-Tips spraying from 80:20 MeCN:H_2_O + 0.1% TFA (v/v) and analyzed using an electrospray ionization mass spectrometer (SYNAPT HDMS).

### Circular dichroism study

The circular dichroism studies were performed at room temperature (25 °C) on *Gaussia* (5 μM), DPS (4 μM) and *Gaussia*-DPS (1 μM). The 20 mM phosphate buffer was used (pH = 8.3). Measurements were performed on Applied Photophysics Chirascan-Plus fitted with a Quantum Northwest temperature controller (JASCO UK).

### Cell culture study

Immortal non-tumorigenic and carcinoma cells were selected for this study: SKBR3 (ATCC® HTB-30™) and MDA-MB-231 (ATCC® HTB-26™) breast cancer epithelial cells, with MRC5 (ATCC® CCL-171™) fetal lung fibroblast cells (all from the American Type Tissue Collection (ATCC)) as the non-tumorogenisis control. These were cultured under optimum conditions in RPMI nutrient medium (Sigma-Aldrich) supplemented with 10% fetal bovine serum (FBS) (Sigma Aldrich), and subcultivated twice weekly to maintain logarithmic growth. MRC5 cells were cultured using Minimum Essential Medium (MEM) supplemented with 10% (v/v) of FBS, L-glutamine (200 mM), non-essential amino acid (10×), HEPES (1 M, pH 7.0-7.6), sodium bicarbonate (7.5%), and penicillin–streptomycin solutions (10,000 U penicillin and 10 mg streptomycin/mL; used at recommended quantity of 10 mL/L) (Sigma Aldrich). For the MTT assay, cells were seeded into 96-well plates at a density of approximately 3 × 10^3^ cells/well and allowed to adhere to the plates by incubating at 37 °C for 24 h in a 5% CO_2_ atmosphere. The cells were treated with Gluc-LiDps-ZnPP at final concentrations between 0.1 μg/mL and 100 μg/mL for 1, 2, 3, 4, 5, or 24 h at 37 °C to allow the uptake of the agent. The medium with excess Gluc-LiDps-ZnPP agent was aspirated and washed twice with 200 μL of PBS. A 200 μL of fresh medium with coelenterazine (5 μg/mL) was added and the cells were further incubated at 37 °C for 72 h in a 5% CO_2_ atmosphere. MTT (Sigma Aldrich), 50 μL (2 mg/mL) was added to each well to give a total volume of 250 μL and incubated for 3 h at 37 °C in a 5% CO_2_ atmosphere. The medium was aspirated. DMSO was used to dissolve the formazan crystals. For studies in 96 well plates, formazan crystals were solubilized with 150 μL of DMSO per well. The absorbance was measured at 550 nm using an En Vision 2104 Multilabel microplate reader (PerkinElmer).

To probe the cellular uptake, cells were seeded at 3.5 × 10^5^ cells/well in a total medium volume of 2 mL in a 6-well plate for 24 h before treatment with 2 mL of Gluc-LiDps-ZnPP (14.8 μg/mL) for 1, 3, and 6 h. The cells were then washed twice with PBS (2 mL), trypsinized (500 μL, trypsin–EDTA 0.25% (w/v) solution, Sigma Aldrich), pooled together with 1 mL of fresh RPMI nutrient medium and centrifuged at 1200 ×*g* for 5 min (Beckman Coulter Allegro). The cell pellet was washed twice with PBS (1 mL), centrifuged and resuspend in 0.5 mL of PBS. Samples were analyzed using an Astrios EQ flow cytometer with excitation at λ = 425 nm and the data were analyzed using Kaluza Flow Cytometry Analysis Software (Beckman Coulter).

The ROS-Glo™ H_2_O_2_ kit (Promega) was used to determine the intracellular ROS levels. Cells were seeded at a density of 5 × 10^4^ cells/well in white 96-well plates and treated after 24 h with 14.8 μg/mL of Gluc-LiDps-ZnPP in the presence of 5 μg/mL coelenterazine. The H_2_O_2_ substrate (20 μL) was mixed with 80 μL of treated cell culture and incubated for 6 h. The detection solution (luciferin detection reagent, D-cysteine and Signal Enhancer Solution (Promega); 100 μL) was added and the cells were incubated for 20 min at room temperature before luminescence detection at (λ = 520 nm) using a microplate reader (En Vision 2104 Multilabel microplate reader (PerkinElmer).

For the wound healing (migration) assay, SKBR3 cells were seeded in a 35 mm μ-Dish containing two medium chambers separated by a membrane (ibidi GmbH, Munich, Germany) at a density of 35 × 10^3^ cells/well and incubated for 24 h. After the membrane was removed, the dish surface was filled with 2 mL of the medium containing 7.4 μg/mL of Gluc-LiDps-ZnPP. The coelenterazine (5 μg/mL) was added after 5 h and the cell migration was followed by tracking the images every 8 h using a Nikon ECLIPSE TS 100 microscope. The images were analyzed using ImageJ (Fiji) software.

### Confocal microscopy studies

Cells were seeded at a density of 1 × 10^4^ cells/well in an 8 well μ-slide confocal chamber (ibidi GmbH, Munich, Germany) and incubated overnight. Following 1, 3 and 6 h treatment with Gluc-LiDps-ZnPP, the cells were washed 3 times with 200 μL of PBS, fixed using 200 μL of 4% (v/v) formaldehyde for 20 min and washed with PBS (3 × 2 μL). The fixed cells were stained with DRAQ5™ DNA stains before washing with 200 μL PBS (3 × 2 μL). Imaging was performed on a confocal microscope (LEICA DMI 4000 B) set at *λ*_*ex*_ = 425 nm and *λ*_*em*_ = 593 nm. The images were analyzed using ImageJ (Fiji) software.

### Statistical analysis

The data were analyzed using one-way, two-way analyses of variance (ANOVAs), and *t* test as appropriate using GraphPad Prism ver. 6. The statistical differences were assessed using the ANOVA method and a significant effect was identified if *P* < 0.05.

## Results

### The construction of Gluc-LiDps-ZnPP

**Figure 1:**
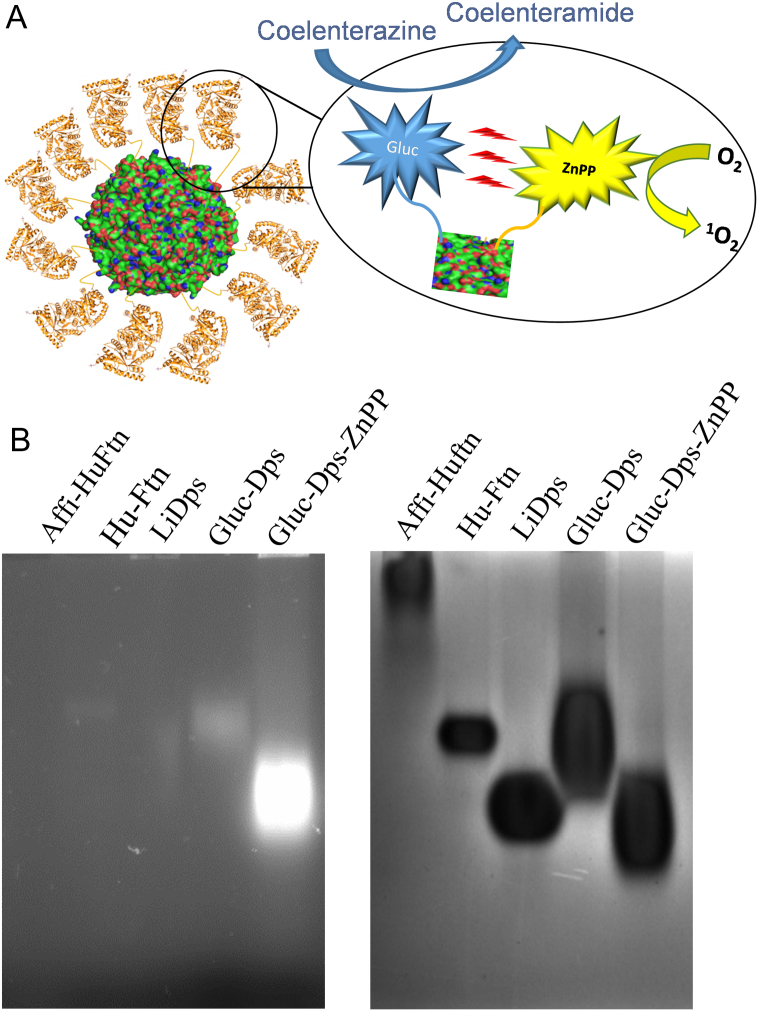
**(A)** Schematic representation of a mechanism of the structure of Gluc-LiDps-ZnPP and generation of ROS. **(B)** Photograph of a native agarose gel under UV illumination (λ_ex_ = 300-400 nm) (left) and stained with Coomassie brilliant blue R-250. Human apo-ferritin (HuAFn) (561 kDa), and LiDps (257 kDa) proteins were used as molecular weight markers after their mass was determined by ESI-MS. Molecular weight of Gluc-LiDps is estimated to be 459 kDa and Gluc-LiDps-ZnPP to be ~515 kDa.

The Gluc-LiDps-ZnPP conjugate was constructed using the natural LiDps gene and a synthetic Gluc gene (with hexa His-tag at the N-terminal and the flexible linker peptide (TGGGGGGLVPRGS) between the proteins). The presence of Gluc-LiDps gene was confirmed by DNA sequencing (Figure SI2). It was found that ~7 lysine residues in each Gluc-LiDps fusion protein could be chemically labeled with ZnPP molecules (Figure 1 and SI1). The fusion protein was expressed in *E. coli* and purified using nickel (II) IMAC and gel filtration. The SDS PAGE gel revealed a band at the expected molecular weight for the Gluc-LiDps construct (theoretical mass = 38.242 kDa), which was also confirmed by nano-electrospray (nESI) mass spectroscopy (measured mass = 38.241 kDa) (Figure SI3). These results, in combination with native PAGE and morphological characterization using TEM (Figures 1, *B* and SI4), confirmed that Gluc-LiDps was isolated in high purity and forms a nanocage of 12 subunits with the diameter expected.

### Bioluminescence of the Gluc-LiDps and BRET of the Gluc-LiDps-ZnPP nanoconstructs

**Figure 2:**
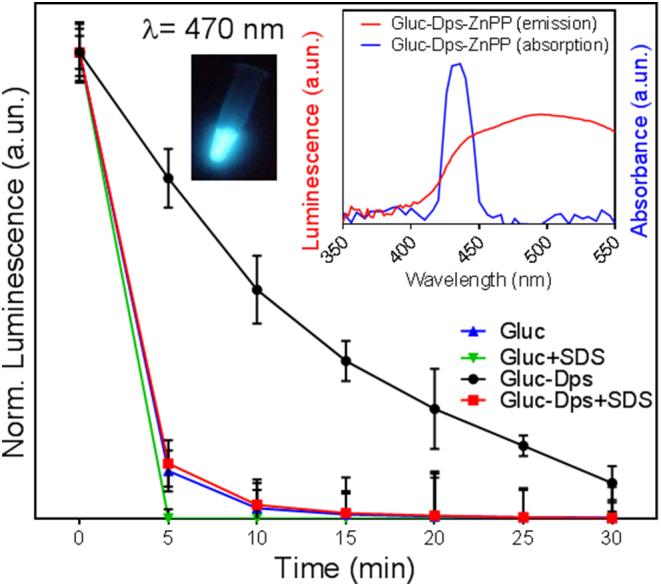
The normalized luminescence intensity measured at *λ* = 470 nm for Gluc-LiDps and for Gluc with and without SDS (normalized to intensity at time 0). Error bars represent average value over three independent repeats. Left inset shows a photograph of blue light emitted by the Gluc-LiDps construct. Right inset shows a spectral overlap between the bioluminescence of Gluc and absorption of ZnPP.

Gluc-LiDps activity was tested by adding the coelenterazine substrate (50 μL at final concentration 4 μg/mL) to the purified protein (100 μL at final concentration 5 μg/mL). Upon addition, blue luminescence light was immediately observed. We found that the light intensity in the presence of Gluc on its own, decayed up to 2× faster compared to that in the presence of the Gluc-LiDps fusion protein (see Figure SI5), and this was independent of coelentrazine concentration (2, 4, and 6 μg/mL). The causes of prolonged lifetime, were probed by addition of 10 μL of sodium dodecyl sulfate to the reaction mixture containing 5 μg/mL of Gluc and 4 μg/mL of coelenterazine. The SDS is commonly used to disassemble multi-subunit proteins,^37^, ^38^ thus reducing steric hindrance for substrates. Hence we used this approach to confirm that activity observed is related to Gluc-LiDps construct and provides additional evidence for its successful formation. We found that after addition of SDS (0.1% w/v) to the reaction mixture (100 μL of final concentration of 5 μg/mL Gluc-LiDps or Gluc both with 4 μg/mL of 50 μL of final concentration of coelenterazine), the fluorescence decay of Gluc-LiDps became comparable to that of Gluc alone (Figure 2).

To achieve an active BRET pair that generates ROS, Gluc-LiDps lysine residues were conjugated with ZnPP pre-activated using EDC/NHS and dialyzed to remove unconjugated ZnPP and products of the coupling reaction. The conjugation efficiency was examined by SDS PAGE and nESI mass spectrometry. We achieved the optimal level of conjugation with a molar ratio of 1:10 for Gluc-LiDps:ZnPP (Figure SI6). The molecular weight of subunits was estimated to be 38,241.7 Da/subunit and increased to 42,938 Da/subunit following ZnPP conjugation. From the combination of nESI-MS results and SDS PAGE, we have estimated that ~7 ZnPP molecules per subunit were attached to the Gluc-LiDps. We note, that the Gluc-LiDps protein precipitated when higher levels of ZnPP (at ratios greater than 1:10 of Gluc-LiDps:ZnPP) were used in the labelling process, probably due to the reduction in polar/ionized amino acids on its surface. Native PAGE studies confirm that the chemically modified proteins formed a protein cage and indicate that Gluc-LiDps-ZnPP migrates faster through the gel compared to Gluc-LiDps, in accord with a change of the net charge (pI) arising from neutralization of some of the positively charged lysine residues after conjugation of ZnPP (Figure 1, *B*).

Inset in Figure 2 illustrates the overlap of luminescence of Gluc and absorption of ZnPP, which is expected to lead to BRET in Gluc-LiDps-ZnPP. Here the bioluminescence energy of the excited coelenterazine generated by Gluc (emission *λ*_*Gluc*_ = 470 nm) is efficiently absorbed by ZnPP (absorption *λ*_*ZnPP*_ = 425 nm).

### Photodynamic effect of Gluc-LiDps-ZnPP on cells

To assess the potential of Gluc-LiDps-ZnPP to act as a PDT agent, we selected two breast cancer cell lines: the triple negative MDA-MB-231 cell line, and the HER2 overexpressing SKBR3 cell line. In addition, the MRC5 fibroblast cell line from fetal lung tissue was included as a non-tumorigenic control to enable differentiation between agents that are general toxins and those which are truly cancer cell line specific. The fibroblast cells are not immortal, they can only divide a set number of times before they senesce and eventually die, however their DNA integrity is maintained providing protection from cancer formation, hence they provide direct comparison for toxicity screens that depend upon proliferation of cells (such as the MTT assay).

The inhibitory growth effects of the Gluc-LiDps-ZnPP in the presence of the coelenterazine were probed using a cell viability (MTT) assay. In order to discriminate between the effects of individual components and that of the nanocage conjugate, cells were exposed to coelenterazine only, Gluc-LiDps and Gluc-LiDps-ZnPP with and without the coelenterazine for 72 h. For coelenterazine alone, no toxic effects were observed following exposure of up to 5 μg/mL of the coelenterazine to the cells, thus this concentration was chosen for further studies (Figure SI7). The maximum exposure concentrations of individual components were selected to correspond to their equivalent concentrations in 100 μg/mL of Gluc-LiDps-ZnPP.

**Figure 3:**
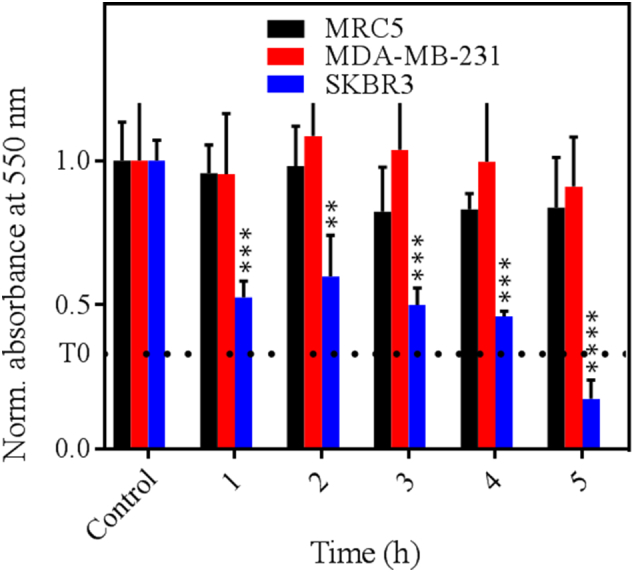
Time dependent growth inhibitory effect of Gluc-LiDps-ZnPP with coelenterazine on SKBR3, MDA-MB-231 and MRC5 cells represented at normalized absorbance at 550 nm (normalized to control). Values are mean ± SD of three independent experiments. Statistically significant differences are labeled as ***P < 0.001 and **P < 0.01.

We found that only exposure of cells to Gluc-LiDps-ZnPP at concentrations >14.8 μg/mL in the presence of coelenterazine (5 μg/mL) led to significant growth inhibition of SKBR3 cells. In contrast, the viability of MDA-MB-231 and MRC5 cells was not affected up to 100 μg/mL (Figure SI8). The time dependent growth inhibition study supported the results of the MTT assays. After 72 h incubation, a clear time-dependent response was observed only in SKBR3 cells and not the other two cell lines (Figure 3).

**Figure 4:**
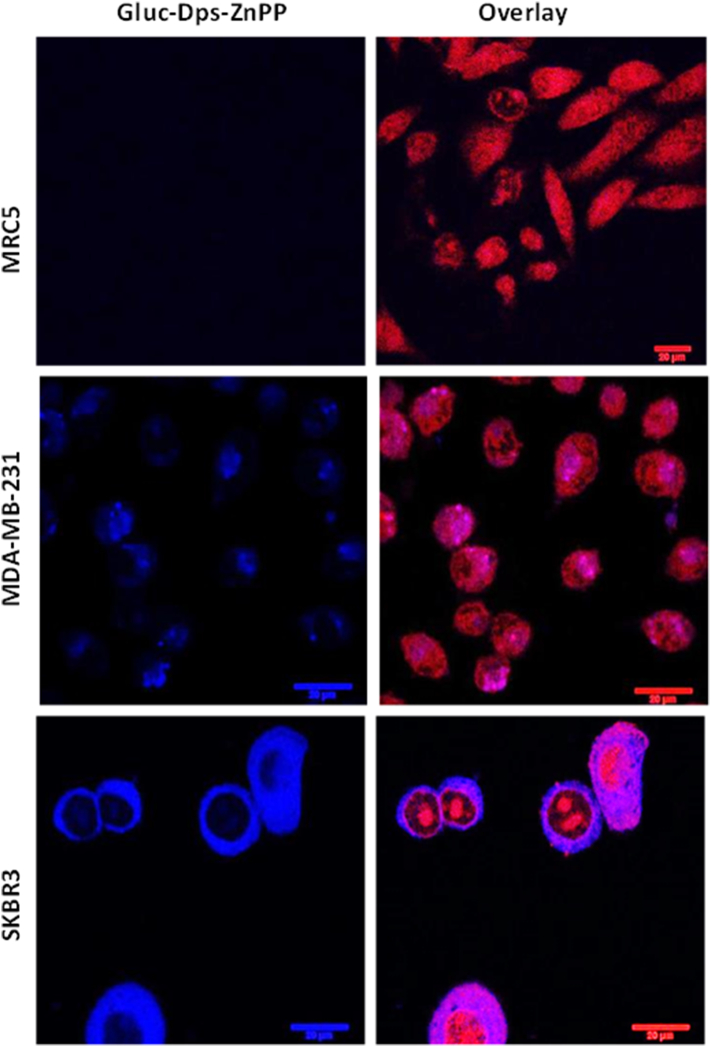
Confocal images of MRC5, MDA-MB-231 and SKBR3 cells demonstrating uptake of Gluc-LiDps-ZnPP after 6 h incubation; blue fluorescence is indicative of the Gluc-LiDps-ZnPP, red fluorescence is indicative of DRAQ5 DNA stain; the overlay of Gluc-LiDps-ZnPP and DRAQ5 in the second column is shown that the Gluc-LiDps-ZnPP is primarily localized in the cytoplasm. Scale bar in all images in 20 μm.

Flow cytometry studies were performed following treatment of the cells with Gluc-LiDps-ZnPP at a concentration of 29.6 μg/mL and demonstrated that most of the Gluc-LiDps-ZnPP was taken up within 3 h of exposure. The cellular uptake by SKBR3 is 2× higher when compared to that with MDA-MB-231 cells (Figure SI9–10 and Table 1 & 2 in SI). Confocal microscopy revealed no detectable emission in the non-tumorigenic MRC5 cells and preferential location of the Gluc-LiDps-ZnPP agent in the cytoplasm of SKBR3 and MDA-MB-231 tumorigenic cells with significantly greater uptake of the Gluc-LiDps-ZnPP agent is observed in the former. (Figures 4 and SI11).

**Figure 5:**
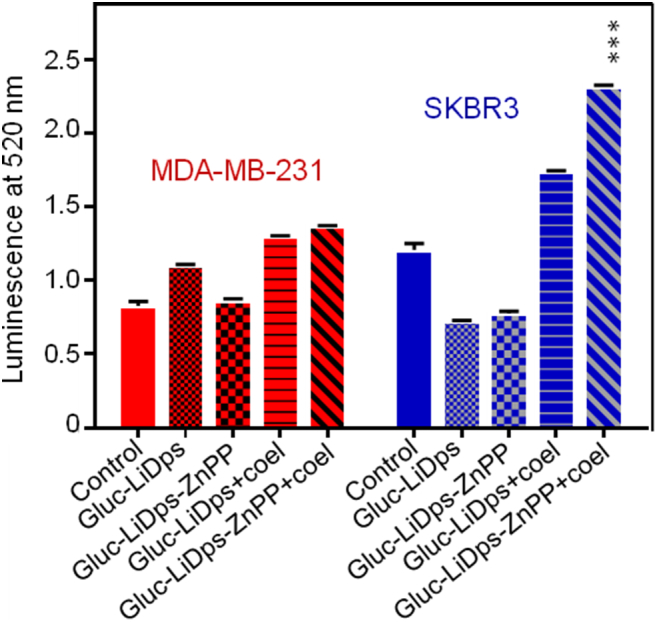
ROS levels generated in SKBR3 and MDA-MB-231 cells treated with Gluc-LiDps-ZnPP and coelenterazine compared to untreated (control) cells. Values are mean ± SD of three independent experiments. Statistically significant differences are labeled as ***P < 0.001.

In order to determine the increase of intracellular ROS caused by the Gluc-LiDps-ZnPP plus coelenterazine, a ROS assay was used. The results show that ROS generation was two-fold higher in SKBR3 cells treated with Gluc-LiDps-ZnPP in the presence of coelenterazine compared to the SKBR3 cells treated separately with each individual compound (Gluc-LiDps-ZnPP, Gluc-LiDps, and ZnPP). The ROS in the MDA-MB-231 cell line was half that observed in the SKBR3 cells (Figure 5) which correlates with the different levels of uptake of the PDT agent in these two cell lines.

**Figure 6:**
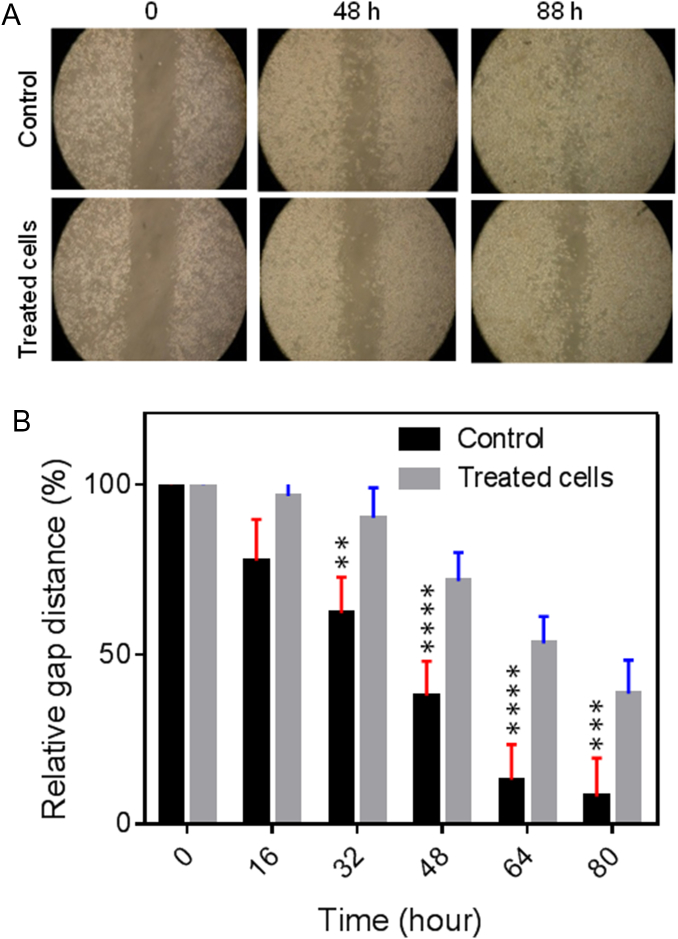
**(A)** The photograph of a wound healing assay performed on SKBR3 cells. The cell migration images tracked immediately after treatment *t* = 0 and every 8 h thereafter. **(B)** The relative change of gap distance with time for SKBR3 cells as a measure of cell migration. Mean values are shown ± SD of three independent experiments.

The effect of Gluc-LiDps-ZnPP plus coelenterazine on SKBR3 cell migration was identified by determining its ability to suppress a ‘scratch’ wound in a layer of cells over time. SKBR3 cells (35 × 10^3^ cells/well) were treated with 2 mL of final concentration of 7.4 μg/mL of Gluc-LiDps-ZnPP for 5 h before adding of 2 mL of final concentration of 5 μg/mL of coelenterazine. The wound was created by a membrane separating the two cell chambers and cell migration was tracked every 8 h. Following 40 h observation, it was evident that exposure to the agent has significantly reduced SKBR3 cell migration compared to untreated SKBR3 cells (Figure 6).

## Discussion

We have constructed the Gluc-LiDps-ZnPP conjugate for the first time and this has the ability to generate ROS *via* BRET. The nanoconstruct retains the structure of the native protein as determined by Circular Dichroism (Figure SI3) and self-assembles into a cage with external diameter ~ 9 nm and the expected molecular weight.^22^ Our studies show that the fusion of Gluc and LiDps significantly enhances the bioluminescence lifetime of Gluc. We attribute the prolonged luminescence of Gluc-LiDps to a decrease in the coelenterazine turnover rate caused by the steric hindrance of the active site of Gluc by LiDps rather than a reduction in the amount of correctly folded protein. This explanation is also supported by further studies demonstrating that the addition of SDS to the reaction mixture, which causes protein disassembly,^37^, ^38^ reverses the observed change of bioluminescence lifetime. This may explain why the fusion of Gluc to LiDps alters the kinetics of the enzyme from ‘flash’, where substrate turnover is very fast and the photoluminescent period is very short^39^ to more ‘glow’ like, where substrate turnover is slower and lasts up to 30 min. Thus incorporation of Gluc into the Gluc-LiDps-ZnPP nanoconstruct offers a benefit of prolonged luminescence lifetime for BRET.

We have studied the effect of the composition of the nanoconstruct on BRET pair efficiency, compound stability and solubility. The optimal ratio of ~7 ZnPP molecules per subunit was found to allow the efficient formation of the protein cage and generate a stable soluble product. As expected from a BRET pair,^13^ following conjugation of ZnPP, the bioluminescence of Gluc at 480 nm^15^ was significantly quenched (> 10-times) compared to that of a mixture of Gluc-LiDps and unconjugated ZnPP at the same concentrations, demonstrating that the BRET process is only efficient in the conjugates, as was previously reported for various BRET systems.^40^

An *in vitro* assessment of the potential of Gluc-LiDps-ZnPP as PDT agent was performed in two breast cancer cell lines (MDA-MB-231 and SKBR3) and in non-tumorigenic MRC5 fibroblast cell line. The cell viability studies confirmed our hypothesis. As expected, the individual constituents have not induced noticeable effects on cell viability and did not induce any change of ROS levels. Only Gluc-LiDps-ZnPP in the presence of coelenterazine led to growth inhibitions, confirning efficient energy transfer from Gluc to ZnPP. Activation of ZnPP results in ROS-generation^20^ leading to cell death. Also, elevated ROS levels were confirmed by the use of a ROS assay for Gluc-LiDps-ZnPP in the presence of coelenterazine. We conclude, that the ROS generation in our nano-construct is due to presence of ZnPP, which is known for its ability to photo-generate singlet oxygen species.^19^ The mechanism of ROS generation in our nanoconstruct remains the same as that of ZnPP, which is ascribed to alterations of cell cycle regulation^41^: upon absorption of photon, the first excited triplet state is formed with long lifetime, the energy is transferred to oxygen forming reactive singlet molecular oxygen.^42^ We also note that the activity of our nanoplatform is comparable to that of ZnPP, indicating that the purification step efficiently removed contamination, and even if negligible amounts of contaminants (*e.g.* lipopolysaccharides) are present, they do not alter PDT activity. The key advantage of our approach is the activation of ROS generation through BRET, *i.e.* without the need for external source of energy, which is beneficial for deep tissue applications.

Interestingly, we find a 2-fold difference in the uptake of the Gluc-LiDps-ZnPP nanoconstruct by SKBR3 cells compared to MDA-MB-231 cells, and negligible levels of uptake by MRC5 cells. We note, that ZnPP alone non-selectively penetrates all studied cells in significantly greater quantities compared to Gluc-LiDps-ZnPP. The observed differences in the uptake are likely due to the presence of specific receptors.^43^ Dominating internalization pathways for ferritin-and Dps-family proteins is through endocytosis, where presence of specific receptors and particular selectivity for TfR1 overexpressing cell lines is widely acknowledged.^44^ Non-selective internalization of these proteins is observed *via* endocytosis through clathrin-coated pits. Further detailed studies are required to confirm the mechanism of internalization. Receptor mediated uptake of fusion protein potentially could offer some selectivity for the delivery and distribution of ZnPP towards cancer cells. In agreement with the observed 2-fold greater uptake is the accompanying 2-fold higher level of ROS in Gluc-LiDps-ZnPP treated SKBR3 cells compared to MDA-MB-231 cells. These results confirm our PDT agent effectively generates ROS and the elevated ROS levels in SKBR3 cells sensitizes these cells to the treatment of Gluc-LiDps-ZnPP with coelenterazine.

Cell migration is an indicator of changes in the cell physiological and pathological processes.^45^ We note, that following exposure to the Gluc-LiDps-ZnPP nanoconstruct affects the ability of cells to migrate, which is of potential benefit for applications in PDT. Further *in vivo* studies are needed to assess the blood clearance time, enhanced permeability and retention (EPR) effect, and the possible immune response of the conjugate which if significant, could be addressed by conjugation of Gluc-LiDps-ZnPP with polyethylene glycol (PEG) to reduce any immune system stimulation.^46^, ^47^ Also, *in vivo* studies will clarify any potential differences between the activity of our nanoplatform and its biodistribution, with particular focus on its interaction with HO-1, which is primarily found in the spleen, liver and kidneys although its level in other cells is raised during stress.

In conclusion we have developed a PDT ‘nanoplatform’ activated *via* BRET with a luciferin (Gluc-LiDps-ZnPP) and demonstrated *in vitro* its ROS generating ability in cancer cells. The conjugate was found to have enhanced uptake and higher cytotoxicity in breast cancer cells (MDA-MB-231; SKBR3) compared to the non-tumorigenic cells examined, demonstrating the significant potential of Gluc-LiDps-ZnPP to be used as a self-illuminating - PDT agent for cancer therapy. This system offers several advantages over the previously reported QD-luciferase/e6-chlorin and luciferase-Rose Bengal BRET/PDT systems including: fewer steps to construct the agent; a completely biodegradable agent that maximizes BRET and hence PDT efficiency by keeping the luciferase and photosensitizer in close proximity; the use of a biocompatible coelentrazine that is known to have good biodistribution and low toxicity *in vivo.* The presence of the hollow cavity of LiDps offers additional opportunities for encapsulation and delivery of drugs and/or imaging agents,^48^ while the exterior surface of the LiDps can be further modified with selective targeting ligands such as antibody fragments or peptides that target receptors overexpressed on tumors.

## Appendix A Supplementary data

Supplementary material Image 1
