# Supplementary material for: Listeria innocua Dps as a nanoplatform for bioluminescence based photodynamic therapy utilizing Gaussia princeps luciferase and zinc protoporphyrin IX
Source: Nanomedicine. 2019 Aug;20:102005. doi: 10.1016/j.nano.2019.04.008 (PMC6712498; doi:10.1016/j.nano.2019.04.008)
Supplement: Supplementary file 1 — Supplementary material [file mmc1.docx]

**Supplementary Information**

*Listeria innocua* Dps as a nanoplatform

for bioluminescence based photodynamic therapy utilizing *Gaussia* luciferase and Zinc protoporphyrin IX

Ali W. Al-Ani, Lei Zhang, Lenny Ferreira, Lyudmila Turyanska,

Tracey D. Bradshaw, Neil R. Thomas

The crystal structure of LiDps protein indicates that the N- terminal of each subunit surrounded by ~28 lysine residues at a distance < 100 Å. Accordingly the fusing of Gluc on the N- terminal of LiDps permits efficient transfer of the energy from Gluc to the surrounding ZnPP molecules and consequently generates of singlet oxygen through BRET (Figure 1).


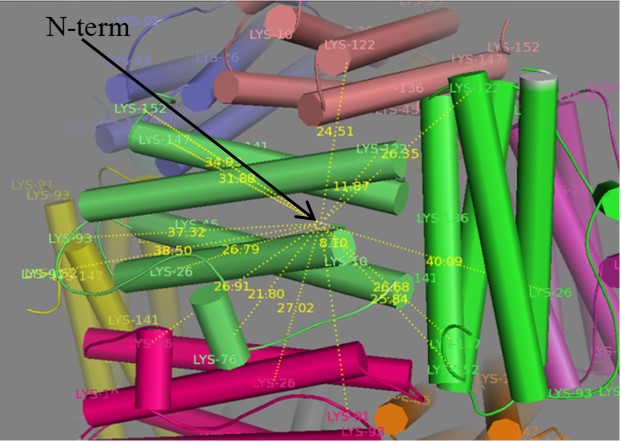


**Figure SI1**. A model of LiDps crystal structure from the Protein Data Bank (PDB ID: 2BKC) created by PyMol. This model shows 28 lysine residues close to the N-terminal at a distance ≤ 100 Å, this distance permits transfer of energy from Gluc (fused in the N-terminal of LiDps) to the ZnPP molecules that are conjugated to lysine residues of Gluc-LiDps via BRET.

Gluc-LiDps gene was designed and inserted in pJexperess an *E. coli* expression plasmid. The gene was characterized by for DNA sequencing (Figure 2).

**Figure SI2**. The map of pJexpress plasmid and the primary amino acid sequence of the Gluc-LiDps chimera. An N-terminal hexa histidine-tag (blue), Gluc enzyme (green), pentaglycine linker with thrombin cleavage site underlined (red) and C-terminal LiDps protein (brown) comprise the sequence.

Recombinant Gluc-LiDps protein was purified using IMAC and size exclusion chromatography. Native PAGE and denatured electrospray mass spectrometry was performed to detect the exact molecular weight of the Gluc-LiDps subunit. The Gluc-LiDps mass spectrum was identical to its theoretical molecular weight whilst the CD spectra of all three proteins indicate that they are all folded, (Figure 3).

**(C)**

**Figure SI3**. **(A)** SDS PAGE electrophoresis of Gluc-LiDps chimera. The band representing Gluc-LiDps subunit was observed at approximately 37 kDa, (the calculated mass is 38241.7 kDa). **(B)** Mass spectrum of Gluc-LiDps chimera, the protein was analyzed using nano- electrospray mass spectrometry after processing through C18 zip tips. The spectrum shows that the molecular weight of Gluc-LiDps subunit is 38 kDa, and matches the theoretical mass of this protein. (**C**) The Circular Dichroism spectra of LiDps (orange line), Gluc (grey line) and LiDps (blue line) recorded on an Applied Photophysics Chirascan-Plus fitted with a Quantum Northwest temperature controller (JASCO UK). The spectra were obtained in 20mm phosphate buffer pH 8.3 at 25 ^o^C using 5 μM of each protein.

TEM imaging was performed to confirm the formation, purity and structure (morphology) of the protein (Figure 4).


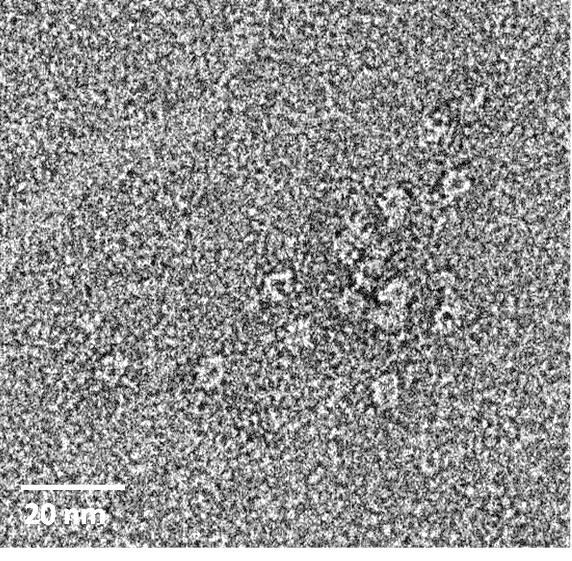


**Figure SI4** TEM image of Gluc-LiDps confirm the formation of the cage. The protein at a concentration of 0.05 mg/mL in 20 mM Tris buffer was deposited on a graphene-coated grid, and TEM images were recorded on the JEOL2100EX microscope operating at 120 kV.

The activity of Gluc was studied after fusing with LiDps using various concentrations of Gluc-LiDps, Gluc, and coelenterazine (the substrate of Gluc). We found that the light intensity of Gluc decayed to ~75% of its initial level compared to < 50% for Gluc-LiDps over the initial 5 minute period (Figure 5 A-B in ESI). At a fixed concentration of Gluc-LiDps (5 µg/mL) and 2 µg/mL coelenterazine, light intensity decreased by 50% after ~ 6 minutes and by 75% after ~ 12 minutes. The observations were very similar for 4 µg/mL and 6 µg/mL coelenterazine respectively. In comparison, the turnover of coelenterazine was very rapid at a fixed concentration of Gluc (5 µg/mL); where the light intensity for 2, 4 and 6 µg/mL coelenterazine is reduced to 0 after 10 minutes (Figure 5 C-D in ESI). These results indicate that Gluc-LiDps possesses significantly longer luminescence activity than Gluc. The activity of Gluc-LiDps displays a ‘glow’ kinetics with gradually decaying bioluminescence, compared to the activity of Gluc alone that displays ‘flash’ kinetics with rapidly declining fluorescence.

**Figure SI5**. Photoluminescence emission at different concentrations of **(A)** Gluc-LiDps (0.5,1, 5, 10 µg/mL) with 10 µM of Coelenterazine (Coel.), **(B)** Glu (0.5, 1, 5, 10 µg/mL) with 10 µM of Coel, **(C)** Coel (5, 10, 15 µM) with 5 µg/mL of Gluc-LiDps and **(D)** Coel (5, 10, 15 µM) with 5 µg/mL of Gluc. Values are mean ± S.D of three independent experiments.

**(A)**

**(C)**

**(B)**

**(D)**

The SDS gel shows the bright bands of Gluc-LiDps protein after conjugation with the ZnPP fluorescent dye. Furthermore the ESI-MS demonstrates that each Gluc-LiDps subunit was labelled with ~ 7 ZnPP molecules. Taken together with mass spectrum interpretation, the results indicate the successful conjugation of ZnPP with Gluc-LiDps using EDC/NHS linking agent.


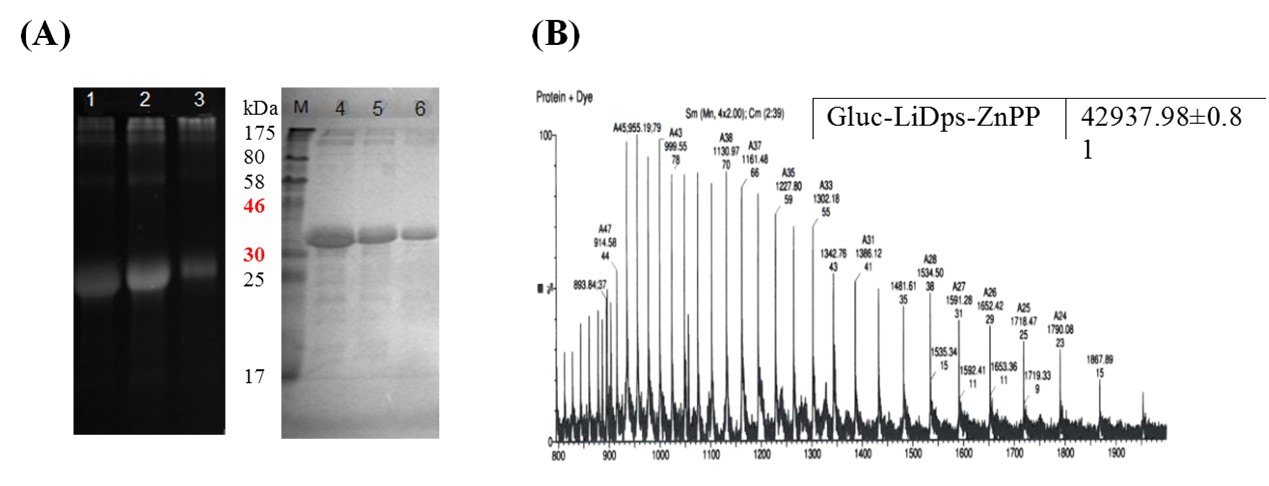
**Figure SI6**. **(A)** SDS gel electrophoresis visualised under UV light and a corresponding gel after staining with Coomassie Brilliant Blue R-250 stain. Lanes 1,2 and 3 correspond to lanes 4, 5 and 6 and represent 1:5, 1:10 and 1:15 molar ratio of Gluc-LiDps:ZnPP respectively. **(B)** Mass spectrum of Gluc-LiDps-ZnPP construct indicates that there are ~ 7 ZnPP molecules bound to each subunit of LiDps. The protein mass spectrum was obtained using nano-electrospray mass spectrometry after processing through C18 zip tips.

The growth inhibitory effect of Gluc-LiDps-ZnPP plus coelenterazine on SKBR3, MDA-MB-231, and MRC5 was studied by MTT assays. Each component of this construct was individually tested. Coelenterazine displayed a non-anti-proliferative effect at 5 µg/mL and this concentration was used in the following growth inhibition studies.

**Figure SI7.** *In vitro* coelenterazine effect assessed by MTT assay. The response of SKBR3, MDA-MB-231 and MRC5 proliferation to coelenterazine doses. Cells were seeded at density of 3 x 10^3^ cells/well in 96-well plates. Values are mean ± S.D of three independent experiments.

Gluc-LiDps-ZnPP plus coelenterazine exhibits strong anti- proliferative activity against SKBR3 cells with 50% of growth inhibition (GI_50_) 14.8 µg/mL compared to MDA-MB-231 and MRC5 cells (GI_50_ > 100 µgmL).

**Figure SI8**. Growth inhibitory effect of GLuc-LiDps-ZnPP plus coelenterazine, GLuc-LiDps-ZnPP, GLuc-LiDps plus coelenterazine, GLuc-LiDps, and ZnPP on the SKBR3 cell line. Cells were seeded at a density of 3 × 103 cells/well in 96-well plates, incubated for 24 hours, and treated with each component for 72 hours. * indicates significant difference compared to control ****(P<0.0001).

**Figure SI9.** The growth inhibitory effects of Gluc-LiDps-ZnPP plus coelenterazine on SKBR3, MDA-MB-231 and MRC5 cell lines.Cells were seeded in 96 well plate at density of 3x10^3^ cell/well. Values are the mean ± S.D of three independent experiments. **** = P < 0.0001.

Cellular uptake of Gluc-LiDps-ZnPP was studied using flow cytometry and its fluorescence detected in SKBR3 was 2-fold greater than that observed in MDA-MB-231 cells; negligible fluorescence was detected in MRC5 fibroblasts. Further flow cytometry results indicate that ZnPP molecules non-selectively penetrated all of these cells in quantities > Gluc-LiDps-ZnPP.

**Figure SI10** Cellular uptake of **(A)** Gluc-LiDps-ZnPP and **(B)** ZnPP by SKBR3, MDA-MB-231 and MRC5 over time was detected using flow cytometry. Cells were seeded at density of 3.5 x 10^5^ cells/well in 6 well plate Values are mean± S.D of three independent experiments. **** = P < 0.0001.

**Table 1** The cellular uptake of Gluc-LiDps-ZnPP construct by SKBR3, MDA-MB-231 and MRC5 in percent.

|  | Cellular uptake of Gluc-LiDps-ZnPP | | | | | |
| --- | --- | --- | --- | --- | --- | --- |
|  | MRC5 | | MDA-MB-231 | | SKBR3 | |
|  | Uptake (%) | SD | Uptake (%) | SD | Uptake (%) | SD |
| 1h | 1.47 | 0.09 | 5.23 | 0.72 | 2.49 | 0.22 |
| 3 h | 1.86 | 0.16 | 28.42 | 0.50 | 35.58 | 2.21 |
| 6 h | 1.67 | 0.14 | 43.56 | 2.31 | 79.85 | 5.25 |

**Table 2** The cellular uptake of ZnPP photosensitizer by SKBR3, MDA-MB-231 and MRC5 in percent

|  | Cellular uptake of ZnPP | | | | | |
| --- | --- | --- | --- | --- | --- | --- |
|  | MRC5 | | MDA-MB-231 | | SKBR3 | |
|  | Uptake (%) | SD | Uptake (%) | SD | Uptake (%) | SD |
| 1h | 1.00 | 0.01 | 4.67 | 0.72 | 2.49 | 0.22 |
| 3 h | 14.78 | 0.69 | 92.61 | 0.29 | 29.01 | 0.16 |
| 6 h | 99.03 | 0.19 | 98.43 | 0.61 | 95.45 | 0.35 |

**Figure SI11**. Flow cytometry histograms where **(A)**, **(B)** and **(C)** represent SKBR3, MDA-MB-231 and MRC5 uptake of Gluc-LiDps-ZnPP respectively, whereas **(D)**, **(E)** and **(F)** represent SKBR3, MDA-MB-231 and MRC5 uptake of ZnPP.

Confocal microscopy images demonstrate that the Gluc-LiDps-ZnPP exhibited cytoplasmic localisation in SKBR3 cells and to a lesser extent in MDA-MB-231 cells, this response was time-dependent. Localisation of Gluc-LiDps-ZnPP could not be detected in MRC5 fibroblasts.

**Figure SI12**. Confocal images of **(A)** SKBR3, **(B)** MDA-MB-231 and **(C)** MRC5 cells, where blue fluorescence is indicative of Gluc-LiDps-ZnPP, red fluorescence is indicative of DRAQ5 DNA stain and the overlay of Gluc-LiDps-ZnPP and DRAQ5 is indicative in the last row of each group. Cells were seeded at density of 1 x 10^4^ cells/well in 8 well plate
